# Low Allergenicity in Processed Wheat Flour Products Using Tannins from Agri-Food Wastes

**DOI:** 10.3390/foods12142722

**Published:** 2023-07-17

**Authors:** Yoko Tsurunaga, Shiori Arima, Sae Kumagai, Eishin Morita

**Affiliations:** 1 Faculty of Human Science, Shimane University, Shimane 690-8504, Japan; 2 Graduate School of Human and Social Sciences, Shimane University, Shimane 690-8504, Japan; 3 Department of Dermatology, Faculty of Medicine, Shimane University, Izumo 693-8501, Japan; emorita@med.shimane-u.ac.jp

**Keywords:** wheat allergy, cookies, tannin, antioxidant activity, quality

## Abstract

The present study aimed to investigate the effect of the addition of tannins from unutilized resources on wheat allergen reduction, antioxidant properties, and quality by substituting 3%, 5%, and 10% of the flour with chestnut inner skin (CIS) and young persimmon fruit (YPF) powders to produce cookies. The enzyme-linked immunosorbent assay and Western blotting showed significantly lower wheat allergen content in CIS- or YPF-substituted cookies than in control cookies, and this effect was pronounced for CIS-substituted cookies. In addition, the tannin content and antioxidant properties of the CIS- or YPF-substituted cookies were markedly higher than those of the control cookies. Quality analysis of the CIS- and YPF-substituted cookies showed that the specific volume and spread factor, which are quality indicators for cookies, were slightly lower in the CIS- and YPF-substituted cookies than in the control cookies. Compared to the control, CIS substitution did not affect the breaking stress and total energy values of the cookies; however, YPF substitution at 10% increased these values. Color was also affected by the addition of CIS and YPF. The results suggest that the addition of CIS and YPF can reduce wheat allergens in cookies and improve tannin content and antioxidant properties.

## 1. Introduction

Wheat is one of the most frequent causes of IgE-mediated food allergies [1]. IgE-mediated food allergies to wheat mostly manifest as wheat-dependent exercise-induced anaphylaxis (WDEIA) in schoolchildren and adults [2]. WDEIA causes allergic symptoms through a combination of secondary factors, such as exercise and nonsteroidal anti-inflammatory drugs, in addition to wheat ingestion, although patients with WDEIA usually ingest wheat products safely without such cofactors. However, the roles of these cofactors have not been well clarified. Furthermore, the amount of ingested wheat increases the risk of induced anaphylaxis [3]. As no immunotherapy or prophylaxis has been established for WDEIA, avoidance of wheat or postprandial rest is recommended, depending on the severity of the allergy symptoms.

A gluten protein, ω5-gliadin, has been identified as a major allergen causing WDEIA [4,5,6,7,8]. As the ω5-gliadin-specific IgE test (ImmunoCAP [f416], Thermo Fisher Scientific, Waltham, MA, USA) has 91% sensitivity and 92% specificity for wheat-related allergic symptoms, WDEIA is also termed ω5-gliadin allergy [3,9]. Although the precise prevalence of ω5-gliadin allergy is unclear, patients with this type of allergy are spread globally. We previously found that the prevalence of ω5-gliadin allergy in adults is 0.021% in Japan [10]. Wheat avoidance, avoidance of combinations of wheat-based meals, exercise, and/or nonsteroidal anti-inflammatory drugs are effective in preventing allergic reactions in most cases of ω5-gliadin allergy. However, standardized prevention measures are not currently established for this immunological disorder [3,11,12]. Moreover, as wheat is used in various commercially available foods due to its high processing characteristics, avoiding wheat in daily meals increases the burden on patients. Therefore, we speculate that reducing the allergenicity of wheat, especially ω5-gliadin, will reduce the risk of WDEIA.

Sensitization to ω5-gliadin has been observed over several years, indicating the difficulty in finding a cure for this disease [13]. Several attempts have been made to reduce the allergenicity of wheat, including epitope degradation using proteolytic enzymes to reduce the allergenicity of wheat, proteolytic cleavage and degradation of wheat gliadins using bacterial and fungal endopeptidases [14], protein denaturation by heat treatment, drying at pasta drying temperatures (20, 60, 110, and 180 °C) with a higher temperature leading to higher protein denaturation [15], and ω5-gliadin deletion wheat line breeding with repeated backcrossing of the 1BS-18 line lacking the gene encoding ω-5 gliadin with elite commercial cultivars [16]. However, these methods have limitations because their effects are not stable or are time-consuming to implement.

Tannins are environmentally friendly, biogenic, natural, and highly reactive polyphenols [17]. Tannins are known to be the second most abundant source of natural aromatic polymers after lignin [18]. Tannins can be divided into two fundamentally different chemical structures: hydrolyzable tannins and condensed tannins. Condensed tannins are compounds in which catechins with flavan skeletons, such as epicatechin and epigallocatechin, are condensed by the C–C bond between C4 and C8 (or between C4 and C6) [19]. Condensed tannins are oligomers and polymers of flavonoid units and require 3–8 flavonoid repeat units to represent a compound as a condensed tannin [17]. Tannins, which have many phenolic groups, interact with a wide variety of different compounds, including many organic species, through physicochemical or physical bonds [17]. In particular, tannins in plants have dramatically high protein-binding capacity and form tannin–protein complexes [20,21].

**Figure 1:**
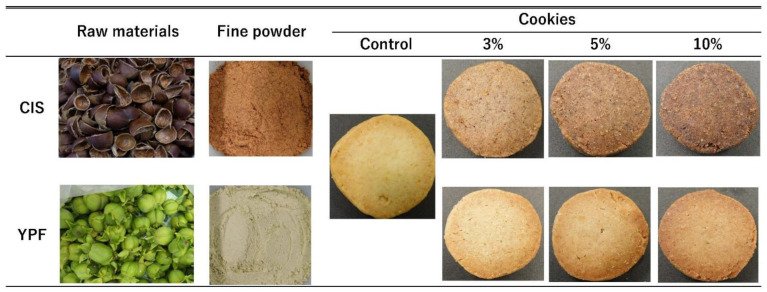
Photographs of the CIS and YPF used as the source of tannin in this study and the cookies produced. CIS, chestnut inner skin; YPF, young persimmon fruit; control, cookies without CIS or YPF; 3%, 5%, and 10%, cookies with wheat flour corresponding to 3%, 5%, and 10% (by weight) of the total ingredient weight (407 g) replaced with CIS or YPF powder, respectively.

Our previous study showed that abundant tannins are present in chestnut (*Castanea crenata* Sieb. et Zucc.) peels and persimmon (*Diospyros kaki* Thunb.) fruits [22,23]. The skin of chestnut fruits comprises the inner skin (CIS) and outer skin (COS) (Figure 1), with CIS having higher tannin content and functionality than COS [22]. CIS also exhibits inhibitory effects on the activity of postprandial glycemic-related enzymes [24]. Chestnuts are roasted, boiled in syrup, or glacé, but a large amount of CIS is discarded during processing due to the peeling process. Young persimmon fruit (YPF) is processed into beverages and juice in Japan and has a high tannin content [25,26]. YPF also has health benefits, such as decreasing blood lipid levels [27] and bile acid binding [28], as well as antibacterial properties [25]. However, a large amount of YPF is wasted due to the thinning operation carried out 2 months before harvest to obtain high-quality mature fruit. Moreover, utilizing these agri-food wastes will also contribute to a sustainable society. As a new application of agricultural food waste, we considered the utilization of the tannin contained in them.

We hypothesized that using tannins to coat IgE-binding epitopes of wheat flour, especially blocking those of ω5-gliadin, will render them hypoallergenic. To prove this hypothesis, we aimed to establish a novel method to block the IgE-binding epitope of ω5-gliadin using CIS and YPF tannins. Addition of tannin may suppress gluten formation, which is important for processed wheat flour products [29]. However, among the wheat-based products, cookies do not require large amounts of gluten; therefore, we speculated that the effect of tannin addition in cookies would less likely affect their quality. Moreover, as cookies contain a high proportion of flour among the total ingredients, their allergen value without tannin would be higher, which may facilitate the evaluation of tannin substitution on allergens. Therefore, we selected cookies as processed wheat products to evaluate the effects of tannin substitution on wheat allergenicity. We used round icebox cookies made with commercial cookie flour as the test material because of their ease of production, uniform quality, and excellent data reproducibility. We also evaluated the effects of tannin substitution on improving the antioxidant properties of the cookies. In addition, we evaluated important cookie quality parameters, including color, spread value, and textural properties, of the tannin-added cookies. This study suggests that the use of CIS and YPF can reduce wheat allergens in cookies and improve tannin content and antioxidant properties.

## 2. Materials and Methods

### 2.1. Cookie Ingredients

In this study, CIS [22] and YPF were selected as tannin-rich materials. To ensure the collection of only the CIS, the ‘Porotan’ cultivar (Tsukuba City, Ibaraki Prefecture, Japan), with excellent peeling ability, was used. First, a slit was made in the peel of the chestnut fruit, and, after being kept in boiling water for 3 min, the peel was separated from the fruit. The peel was then separated into the outer and inner skin, and only the inner skin was dried at 60 °C for 12 h using a constant air temperature oven (DN-61, Yamato Scientific Co., Tokyo, Japan). Young fruits of the ‘Saijo’ cultivar of persimmon were collected from the Shimane Agricultural Technology Center (Izumo City, Shimane, Japan) and were freeze-dried after removing the calyces and seeds. Each sample was crushed using an Oster blender (Osaka Chemical Co., Ltd., Osaka, Japan) and sieved through a 1.0 mm sieve. Samples were sealed in aluminum packs and stored at −25 °C until analysis (Figure 1).

### 2.2. Cookie Production Method

The ingredients in the control cookies were 200 g of wheat flour (cake flour type, Nisshin Seifun Group Inc. Ltd., Tokyo, Japan), 120 g of unsalted butter, 60 g of granulated sugar, 25 g of eggs, and 2 g of salt. To produce the tannin-substituted cookies, the flour was replaced with 3% (12.21 g, corresponding to 3% of total ingredient weight), 5% (20.35 g), and 10% (40.7 g) CIS or YPF powders for a total weight of 407 g. To prepare the cookies, melted butter and salt were mixed in a mixer (DB-2263, Kai Co., Ltd., Tokyo, Japan) at level 2 speed for 30 s, and then granulated sugar was added and mixed at level 6 speed for 5 min. The beaten eggs were added and mixed for 2 min, and finally, the sifted wheat flour with or without CIS/YPF was added and mixed at level 2 speed for 10 min. After wrapping in plastic wrap and refrigerating for approximately 30 min (at 10 °C), the dough was divided into 100 g bars 2 cm in diameter and 16 cm in length, and then wrapped again in plastic wrap and frozen (at −25 °C). The frozen dough was cut into a total of 30 slices (6 mm thick) and baked at 170 °C for 15 min in a steam convection oven (NE-BS1600, Panasonic Inc., Tokyo, Japan) while rotating the baking sheet clockwise every 3 min 45 s to ensure even baking (Figure 1).

### 2.3. Evaluation of Wheat Allergen Content

#### 2.3.1. Enzyme-Linked Immunosorbent Assay (ELISA)

Two ELISA kits, FASPEK ELISA II^®^ series for gliadin (Morinaga Institute of Biological Science, Yokohama, Japan) and FASTKIT ELISA Ver. III^®^ series for wheat (NH Foods Ltd., Ibaraki, Japan), were used in this study to detect wheat allergen content. Both ELISA kits were introduced as an official analytical method in Japan in 2002 [30]. FASPEK KIT II ^®^ uses polyclonal antibodies to detect specific purified proteins or individual proteins of specific components. In wheat, gliadin is targeted [30]. FASTKIT ELISA Ver. III^®^ has the feature of detecting the entire allergen protein using polyclonal antibodies against multiple antigens [30]. In both cases, analytical methods followed the instruction manual provided with each kit [31,32]. The analysis manuals for both methods stated that 19 mL of the extract should be added per gram of sample; however, in our case, the protein content derived from the antigen in the sample was substantially high, which could lead to insufficient extraction. Therefore, we added 19.9 mL of extract to 0.1 g of the sample. As the wheat protein content in the samples was expected to be too high to be evaluated appropriately, the samples were diluted accordingly with extraction buffer to ensure that the concentration was within the measurement range before analysis.

#### 2.3.2. Western Blot Analysis

The levels of ω5-gliadin in the samples were detected using Western blotting as previously described [10]. Cookie samples were dissolved in a sample buffer and heated at 95 °C for 5 min. To determine the protein concentration in the cookie samples, proteins were extracted using the RC DC Protein Assay Kit (Bio-Rad Laboratories, Hercules, CA, USA) to remove β-mercaptoethanol. The concentration was determined following the Lowry method using the DC Protein Assay Kit (Bio-Rad Laboratories). Commercially available wheat flour was separated into water-soluble and water-insoluble fractions. ω5-gliadin was purified from the water-insoluble fraction as described previously [4]. Samples were separated using sodium dodecyl sulfate polyacrylamide gel electrophoresis (SDS-PAGE) on 12.5% acrylamide gels. Proteins were visualized with Coomassie brilliant blue (CBB) staining. For immunoblotting, proteins were electrophoretically transferred to a polyvinylidene difluoride membrane (Immobilon-P; MilliporeSigma, St. Louis, MO, USA) and reacted with polyclonal rabbit anti-ω5-gliadin IgG antibody [16]. The ω5-gliadin bound with the anti-ω5-gliadin IgG was visualized using ECL Prime Western Blotting Detection Reagents (Amersham, Buckinghamshire, UK) after reacting with horseradish peroxidase-conjugated donkey anti-rabbit IgG (GE Healthcare, Buckinghamshire, UK).

### 2.4. Soluble Tannin Content (STC) and Antioxidant Activity Assay

Folin–Ciocâlteu reagent solution (2 N), 1,1-diphenyl-2-picrylhydrazine (DPPH, 95%) (powder), Trolox (97%), 2,2′-azobis (2-amidinopropane) dihydrochloride (AAPH, 95%) (powder), and ethanol solution (99.5%) were purchased from Wako Chemicals Ltd. (Osaka, Japan); fluorescein sodium salt (1 mg/mL in pure water) was purchased from Sigma-Aldrich (St. Louis, MO, USA). Catechin (CTN) (>98%) in a powdered form was purchased from Funakoshi Corporation (Tokyo, Japan). CIS, YPF, and cookies were extracted in 60% ethanol at 40 °C for 2 h with shaking following the protocols described in previous studies [22,33]. The STC of the extracts was measured using the Folin–Ciocâlteu method [34]. Since chestnuts and persimmons contain few low-molecular-weight polyphenols, the Folin–Ciocâlteu method, which is usually used for polyphenol analysis, can be used for STC measurements [35,36,37]. Therefore, the Folin–Ciocâlteu method was also used for STC measurement in this study.

The STC was expressed in terms of mg equivalent/100 g, using CTN as the standard (mg CTN eq/100 g). The antioxidant activity of the extracts was analyzed using the DPPH radical-scavenging assay [33] and hydrophilic oxygen radical absorbance capacity (H-ORAC) assay [38]. The DPPH and H-ORAC values were expressed in terms of µmol Trolox equivalent/g (µmol TE/g).

### 2.5. Cookie Quality Assessment

#### 2.5.1. Appearance and Color

A digital camera (WG-40 W, Ricoh Co., Ltd., Tokyo, Japan) was used to observe the appearance of cookies. The color of the cookies was measured in three categories: L*, black to white; a*, red to green; b*, yellow to blue. L*, a*, and b* values for the peel pastes were measured using a spectrum color reader (CR-13, Konica Minolta, Tokyo, Japan). The results are expressed as the mean ± standard error (SE; *n* = 10).

#### 2.5.2. Specific Volume

The weight of the cookies was measured using an electronic balance, and the volume was calculated using the rapeseed displacement method. The specific volume of the cookie was calculated by dividing the cookie volume by the cookie weight. Three measurements were taken per sample, and the average value was calculated [39,40].

#### 2.5.3. Spread Factor

The spread factor (*n* = 10) was determined by dividing the diameter (mm) by thickness (mm) [39,41].

#### 2.5.4. Textural Properties

The textural properties of the samples were measured using an RE2-33005B Creep Meter (YAMADEN Co., Ltd., Tokyo, Japan). The breaking stress and total energy were measured using a wedge plunger (No. 49; YAMADEN Co., Ltd.) with a velocity of 5 mm/s and a distortion rate of 50%. Ten cookie samples were assessed in each sample group. Measurements were obtained using a 20 N load cell at 20 ± 1 °C. The breaking stress (Pa), maximum stress, and total energy (J/m^3^) of each sample were determined using texture analysis software (Ver. 2.2; YAMADEN Co., Ltd.).

### 2.6. Statistical Analysis

Data were statistically analyzed using SPSS version 28.0 (SPSS Inc., Chicago, IL, USA). Results are expressed as the mean ± SE. Data were tested using one-way analysis of variance (ANOVA), followed by Tukey’s test for multiple comparisons. A *p*-value <0.05 was considered statistically significant.

## 3. Results and Discussion

### 3.1. Wheat Protein Contents Evaluated Using ELISA

**Figure 2:**
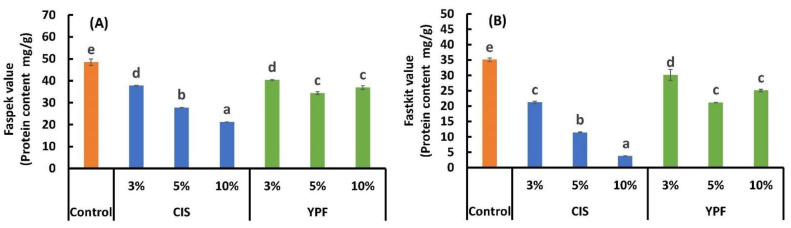
Effect of the addition of CIS or YPF on the wheat protein content in the cookies. Wheat protein contents were assayed using Faspek (**A**) and Fastkit (**B**). CIS, chestnut inner skin; YPF, young persimmon fruit; Faspek, FASPEK ELISA II^®^ series for gliadin, ELISA Kit for single antigen system; Fastkit, FASTKIT ELISA Ver. III^®^ series for wheat, ELISA Kit for composite antigen systems. All results were obtained using Tukey’s test for multiple comparisons. Different letters indicate significant differences at *p* < 0.05. Data are expressed as the mean ± SE (*n* = 3).

The results of the FASPEK ELISA II^®^ series for gliadin (hereinafter referred to as Faspek) and FASTKIT ELISA Ver. III^®^ series for wheat (hereinafter referred to as Fastkit) are shown in Figure 2. In Japan, five major allergen components (egg, milk, wheat, buckwheat, and peanut) require mandatory labeling; the stable analysis values of Faspek and Fastkit, which have been cross-analyzed by 10 research institutions [42], were used as the official screening standards. In our study, both kits showed similar trends, with significantly reduced values in all treatments with CIS and YPF compared to the control. Moreover, the Faspek and Fastkit values remarkably decreased as the addition ratio of CIS increased. In contrast, the addition of YPF decreased the Faspek and Fastkit values, but not in a concentration-dependent manner. The comparison of the effect of adding CIS and YPF on the Faspek and Fastkit values revealed that the effect of CIS replacement tended to be higher. This may be related to CIS and YPF STC. The STCs for CIS and YPF were 26,454 ± 1262 and 3756 ± 175 mg CTN eq/100 g, respectively, which were remarkably higher for CIS (Table 1). Therefore, CIS, which has a high STC, may have bound more antigenic proteins in the reagent than YPF, leading to lower Faspek and Fastkit values. In particular, the Faspek and Fastkit values were significantly lower in the 10% CIS substitution group, with a Faspek value of 48.4 mg/g for the control compared to 21.2 mg/g for the 10% CIS substitution group (43.7% reduction). The Fastkit value was higher for the control (35.0 mg/g) than that for the 10% CIS substitution group (3.7 mg/g; 90.7% reduction) (Figure 2A,B). The more pronounced effect of CIS addition detected using the Fastkit compared to that detected by the Faspek kit could be attributed to the differences in their target antigens. The compound antigen system, Fastkit, showed a more pronounced decrease than Faspek with the addition of CIS and YPF. Studies examining the reaction of tannins with wheat gluten have reported that high-molecular-weight glutenin subunits bind preferentially to the largest available protein fractions, such as ω-gliadin over α-gliadin or γ-gliadin, rather than low-molecular-weight glutenin [43]. This finding suggests that tannins derived from CIS and YPF bind not only to gliadin but also to multiple antigens present in wheat, thus explaining the more pronounced effect of the addition of CIS and YPF with Fastkit than with Faspek. The binding mechanism between proteins and tannins has been elucidated in several studies [44,45,46]. It has been reported that proline-rich proteins and polymers have high tannin binding capacity to tannins, that the binding of tannins to proline-rich proteins involves face-to-face stacking of aromatic groups onto proline residues, and that the interaction with globular proteins involves only surface-exposed aromatic residues [44]. It has also been suggested that the binding of wine tannins to salivary proteins may be governed by hydrogen bonds between the carbonyl functional group of the proline residue and both phenolic and catechol OH groups [45]. Gluten proteins composed of glutenin and gliadin are rich in proline and glutamine [46], and the IgE epitopes of ω5-gliadin and high-molecular-weight glutenin subunits contain proline; thus, tannins may form a strong complex with these proteins.

### 3.2. SDS-PAGE Analysis and ω5-Gliadin Detection Using Immunoblotting

**Figure 3:**
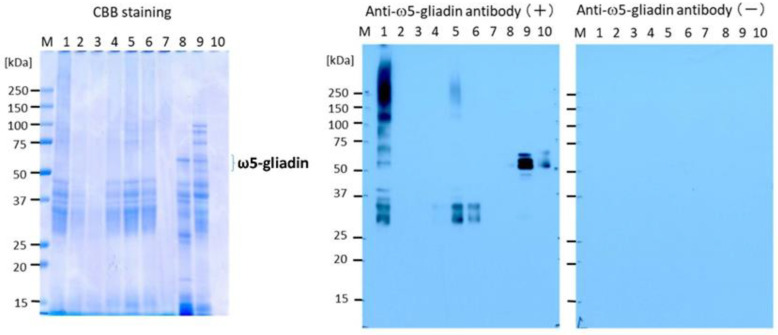
Sodium dodecyl sulfate polyacrylamide gel electrophoresis (SDS-PAGE) analysis of cookie samples and detection of ω5-gliadin using immunoblotting. Lane M, Molecular weight marker; lane 1, control; lane 2, CIS3%; lane 3, CIS5%; lane 4, CIS10%; lane 5, YPF3%; lane 6, YPF5%; lane 7, YPF10%; lane 8, commercial wheat (soluble fraction); lane 9, commercial wheat (insoluble fraction); lane 10, purified ω5-gliadin. CIS, chestnut inner skin; YPF, young persimmon fruit.

The results of immunoblotting of cookie samples for the detection of ω5-gliadin are shown in Figure 3. CBB staining of the cookie samples in the SDS-PAGE gels showed that cookie 1 (control with no tannin) had several protein bands with molecular weights ranging from 25 to 250 kDa in addition to smear staining ranging from the 25 kDa area to the large-molecular-weight area. In contrast, no clear bands were visible in the area of approximately 55 kDa corresponding to ω5-gliadin. The bands in the area between 25 and 250 kDa became faint for cookies 2 (CIS3%), 3 (CIS5%), and 7 (YPF10%). Blotting with anti-ω5-gliadin IgG antibody revealed that immunoreactive ω5-gliadin was widely distributed from the area of molecular weight of 30 kDa to the large-molecular-weight area of over 250 kDa in cookie 1, which could be attributed to polymerized or aggregated gluten proteins as shown in lane 1 (Figure 3). In the blotting against the tannin-treated cookies (samples 2 (CIS3%), 3 (CIS5%), 4 (CIS10%), and 7 (YPF10%)), no reaction to the immunoreactive ω5-gliadin was detected, although weak reactions were observed in cookies 5 (YPF3%) and 6 (YPF5%). These results indicate that the addition of CIS and YPF reduced ω5-gliadin compared to the control, and this effect was pronounced for CIS. These results are well compatible with those of ELISA. Moreover, the reduction in the binding of anti-ω5-gliadin IgG antibodies to the ω5-gliadin proteins is likely due to the competitive blocking of epitopes by tannins in CIS and YPF against antibody binding.

In this study, we confirmed the hypoallergenicity caused by tannin addition using ELISA, SDS-PAGE analysis, and ω5-gliadin detection using immunoblotting. In the future, it will be necessary to examine the binding mechanism of tannins to proteins and how the tannin–protein binding is altered during digestion and absorption.

### 3.3. STC and Antioxidant Activity

**Figure 4:**
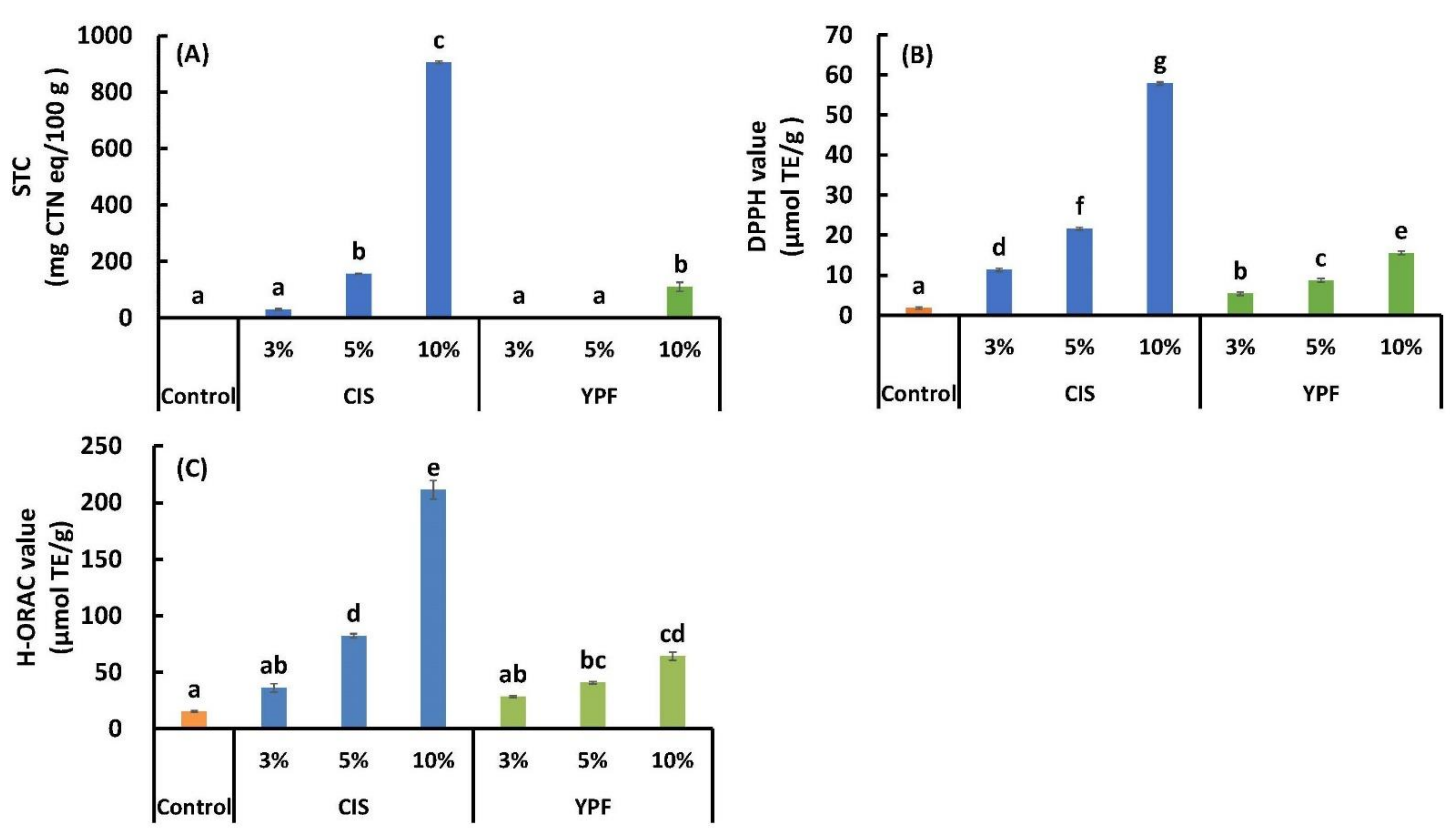
Effect of CIS or YPF addition on the (**A**) STC, (**B**) DPPH, and (**C**) H-ORAC values of cookies. All results were obtained using Tukey’s test for multiple comparisons. Different letters indicate significant differences at *p* < 0.05. Data are expressed as the mean ± SE (*n* = 6). STC, soluble tannin content; CTN, catechin; DPPH, 1,1-diphenyl-2-picrylhydrazine; H-ORAC, hydrophilic oxygen radical absorbance capacity; TE, Trolox equivalent; CIS, chestnut inner skin; YPF, young persimmon fruit.

The values of STC, DPPH, and H-ORAC are shown in Figure 4. STC values at 5% and 10% CIS substitution and 10% YPF substitution were significantly higher than those in the control (*p* < 0.05). DPPH values were significantly higher in all CIS and YPF replacement treatments than that in the control (*p* < 0.05). H-ORAC values were significantly higher at both 5% and 10% CIS and YPF substitution than in the control (*p* < 0.05). These results may be attributed to the raw materials CIS and YPF (Table 1). Although this experiment aimed to reduce allergies by adding tannins, the addition of CIS and YPF also resulted in cookies with higher tannin content and antioxidant properties. In particular, the CIS substitution resulted in more pronounced STC and antioxidant activity (Figure 4).

### 3.4. Cookie Quality

#### 3.4.1. Color and Appearance

**Figure 5:**
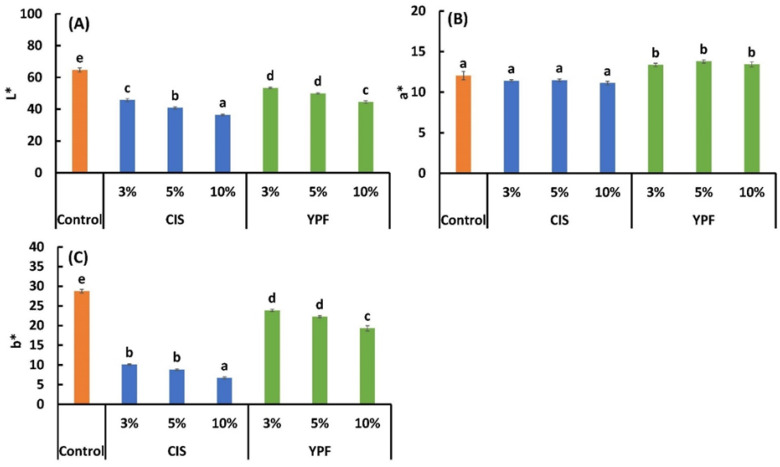
Effect of CIS and YPF addition on the (**A**) L*, (**B**) a*, and (**C**) b* values of the cookies. All results were obtained using Tukey’s test for multiple comparisons. Different letters indicate significant differences at *p* < 0.05. Data are expressed as the mean ± SE (*n* = 10). CIS, chestnut inner skin; YPF, young persimmon fruit.

As shown in Figure 1, the addition of CIS resulted in a darker color than that of the control, which was more pronounced as the substitution ratio increased. In contrast, the addition of YPF did not significantly change the appearance as the substitution ratio increased, and the color became slightly reddish. The L* and b* values of both CIS and YPF decreased as the amount of additives increased, with CIS showing a larger decrease than YPF (Figure 5A,C). The a* value, indicating redness, was not significantly different for CIS-substituted cookies compared to the control, whereas the a* value was significantly higher for YPF (Figure 5B). The L*, a*, and b* values of the raw material CIS were 45.9 ± 0.1, 17.7 ± 0.1, and 16.4 ± 0.1, respectively, and those for YPF were 76.5 ± 0.2, 0.2 ± 0.0, and 17.2 ± 0.1, respectively. The darker color of the cookies with CIS is presumably due to the lower L* and b* values of the raw material CIS, reflecting the color of the raw powder. For a* values, the YPF-substituted cookies had significantly higher values than the control and CIS-substituted cookies. The a* values of YPF in the raw material were significantly lower than those of CIS, resulting in different results for the raw material (Table 1) and cookies (Figure 5). As YPF is a young fruit, the sugar content is presumed to be high [47]. Therefore, the reaction between the sugar derived from YPF in cookies and amino acids in butter and flour may have promoted the aminocarbonyl reaction [48], thereby increasing the a* value.

#### 3.4.2. Specific Volume and Spread Factor

**Figure 6:**
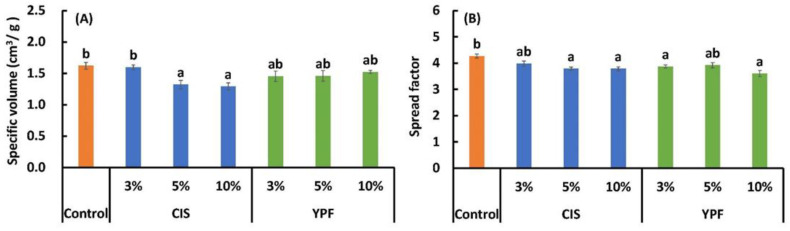
Effect of CIS and YPF addition on the (**A**) specific volume and (**B**) spread factor of cookies. All results were obtained using Tukey’s test for multiple comparisons. Different letters indicate significant differences at *p* < 0.05. Specific volume data are expressed as the mean ± SE (*n* = 3); spread factor data are expressed as the mean ± SE (*n* = 10). CIS, chestnut inner skin; YPF, young persimmon fruit.

The specific volume and spread factor of cookies are shown in Figure 6. The specific volume was significantly lower for 5% and 10% of CIS-substituted cookies (1.32 ± 0.1 and 1.29 ± 0.1 cm^3^/g, respectively) compared to the control cookies (1.62 ± 0.1 cm^3^/g). The specific volume of 10% CIS-substituted cookies with the lowest value (1.29) was 0.8 times that of the control cookies (1.62), whereas no difference was observed between the YPF-substituted and the control cookies.

The spread values were significantly lower for the 5% and 10% CIS and 3% and 10% YPF substitutions than the control. The spread value (3.60) for 10% YPF substitutions was 0.8 times that of the control (4.3). These results demonstrate that the addition of CIS and YPF lowered the specific volume and spread values of cookies. The spread value, the ratio of cookie diameter to spread, is used to predict product quality [41]. Cookies with spread values that are too high or too low cause problems in industrial production, resulting in products that are small in size and very high weight [49]. Therefore, previous studies have focused on the effects of adding byproducts from food processing to cookies. Researchers [39] showed that cookies made from orange byproduct flour had a lower specific volume at higher addition concentrations, suggesting that the interaction between fiber and gluten may reduce the ability of the dough to hold air. Another study [50] reported that the specific volume, volume index, width, thickness, and spread ratio decreased with increasing amounts of citrus dietary fiber preparations. Researchers [49] also reported that, when a mixture of brown rice flour (70%) and cornstarch (30%) is replaced with buriti endocarp flour (0%, 5%, 10%, 15%, or 20%), the spread ratio and specific volume decrease with concentration. As the CIS and YPF used in the present study are thought to be high in dietary fiber and also contain high levels of tannins, which bind tightly to proteins [22,51], gluten formation was significantly suppressed, which may have reduced the ability of the dough to hold air. In the future, we plan to investigate processing methods that retain hypoallergenicity and do not degrade quality, such as making CIS and YPF into a fine powder, adding them as an extract, and identifying the timing of their addition.

#### 3.4.3. Textural Properties

**Figure 7:**
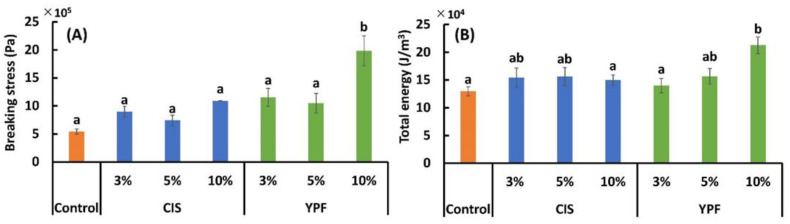
Effect of CIS and YPF addition on the (**A**) breaking stress and (**B**) total energy of cookies. All results were obtained using Tukey’s test for multiple comparisons. Different letters indicate significant differences at *p* < 0.05. Data are expressed as the mean ± SE (*n* = 10). CIS, chestnut inner skin; YPF, young persimmon fruit.

The results of breaking stress (Pa) and total energy (J/m^3^) are shown in Figure 7A,B, respectively. The mean values of breaking stress were higher in both CIS- and YPF-substituted cookies than in the control cookies; however, statistically significant differences were only observed with 10% YPF substitution. Breaking stress in the control and YPF10% zone was 542,480 ± 45,284 and 1,982,193 ± 268,425 Pa, respectively, with the YPF10% zone showing a value 3.7 times higher than that of the control. The total energy results were also similar as the values for both CIS and YPF were higher than those of the control; however, statistically significant differences were only observed in the 10% YPF cookies. The total energy of the control and YPF10% was 129,464 ± 8152 and 212,644 ± 14,977(J/m^3^), respectively; the total energy of YPF10% was 1.6 times higher than that of the control. Tannins in CIS and YPF strongly bind to proteins to form tannin–protein complexes [51], which may have disrupted the gluten network and formed tannin–protein complexes, altering the textural properties of the cookie and resulting in a harder cookie. However, YPF resulted in harder cookies than CIS, suggesting that CIS is particularly high in fiber [22], which may have significantly inhibited the gluten network in the cookies and made them more likely to fall apart.

## 4. Conclusions

In this study, cookies were produced by replacing 3%, 5%, and 10% of the total ingredients with CIS and YPF powders. CIS or YPF substitution significantly reduced the wheat allergenic protein values and ω5-gliadin contents compared to the control, indicating that tannins from CIS or YPF reduce the hypoallergenicity of wheat in cookies. Furthermore, tannin substitution increased the STC and antioxidant properties of cookies compared to the control, wherein it decreased the specific volume and spread factor, key quality indicators of the cookies. However, stress at break and total energy values were not affected by CIS substitution compared to control, whereas YPF showed predominantly higher values at 10% substitution, suggesting a tendency for the cookies to harden. The color appeared to be affected by the color of the added CIS and YPF. In summary, cookies substituted with CIS and YPF powders showed a change in quality depending on the rate of substitution. This study suggests that the addition of CIS and YPF reduces the allergenicity of cookies and improves their STC and antioxidant properties. However, to validate these results, it is necessary to demonstrate the effect of reducing the allergenicity of this substitution method through clinical trials in humans in the future. Although the patients’ sensitivity to gluten protein is very high, we were unable to reduce allergen content to zero. In the future, we plan to examine the cookie production method and the wheat used more closely to further lower allergenicity. Furthermore, to verify the mechanism of the hypoallergenic effects of tannins, we believe that adding fractionated and purified tannin will also be effective. In addition, no sensory analysis was conducted in this study, which is important in terms of cookie quality. Therefore, future studies are necessary to investigate how the addition of tannin changes the taste of the cookies.

## Figures and Tables

**Table 1:** **STC** and color of CIS, YPF powder, and wheat flour.

	STC (mg CTN eq/100 g)	L*	a*	b*
CIS	26,454 ± 1262	45.9 ± 0.1	17.7 ± 0.1	16.4 ± 0.1
YPF	3756 ± 175	76.5 ± 0.2	0.2 ± 0.0	17.2 ± 0.1
Wheat flour (cake flour type)	0	95.2 ± 0.1	0.3 ± 0.0	7.8 ± 0.1

Data are expressed as the mean ± SE (*n* = 6); STC, soluble tannin content; CTN, catechin; CIS, chestnut inner skin; YPF, young persimmon fruit.

## Data Availability

The data that support the findings of this study are available from the corresponding author upon reasonable request.

## Abbreviations

CBB, Coomassie brilliant blue; CIS, chestnut inner skin; CTN, catechin; DPPH, 1,1-diphenyl-2-picrylhydrazine; ELISA, enzyme-linked immunosorbent assay; H-ORAC, hydrophilic-oxygen radical absorbance capacity; SDS-PAGE, sodium dodecyl sulfate polyacrylamide gel electrophoresis; TE, Trolox equivalent; STC, soluble tannin content; WDEIA, wheat-dependent exercise-induced anaphylaxis; YPF, young persimmon fruit.
